# Research of Probability-Based Tunneling Magnetoresistive Sensor Static Hysteresis Model

**DOI:** 10.3390/s21227672

**Published:** 2021-11-18

**Authors:** Yutao Li, Liliang Wang, Hao Yu, Zheng Qian

**Affiliations:** 1 School of Instrumentation and Optoelectronic Engineering, Beihang University, Beijing 100191, China; liyutaobd@163.com (Y.L.); wangliliang@buaa.edu.cn (L.W.); 2 High Voltage Research Institute, China Electric Power Research Institute, Beijing 100192, China; yuhao@epri.sgcc.com.cn

**Keywords:** tunneling magnetoresistive (TMR), hysteresis, Preisach model, probability hysteresis model

## Abstract

Tunneling magnetoresistive (TMR) sensors have broad application prospects because of their high sensitivity and small volume. However, the inherent hysteresis characteristics of TMR affect its applications in high accuracy scenarios. It is essential to build a model to describe the attributes of hysteresis of TMR accurately. Preisach model is one of the popular models to describe the behavior of inherent hysteresis for TMR, whereas it presents low accuracy in high-order hysteresis reversal curves. Furthermore, the traditional Preisach model has strict congruence constraints, and the amount of data seriously affects the accuracy. This paper proposes a hysteresis model from a probability perspective. This model has the same computational complexity as the classic Preisach model while presenting higher accuracy, especially in high-order hysteresis reversal curves. When measuring a small amount of data, the error of this method is significantly reduced compared with the classical Preisach model. Besides, the proposed model’s congruence in this paper only needs equal vertical chords.

## 1. Introduction

TMR is an emerging commercial magnetoresistive sensor with good performance. It has a broad application prospect in automobiles, electronic storage, biomedicine, and aerospace. Hysteresis is a characteristic widely present in magnetic materials, which has a significant impact on the features of the sensor. On one side, the hysteresis characteristic affects the measurement accuracy in specific applications; on the other side, the hysteresis could be utilized for data storage.

Although TMR has been extensively studied, the research on the hysteresis of TMR is still on the way. Current investigations on TMR mainly focuses on its performance, while there is relatively little research on its magnetic hysteresis. There are two main types of research on hysteresis. Portugal INESC [1], Mark Tondra and James M. Daughton [2] improved the hysteresis characteristics of GMR by adding a bias magnetic field; Jean Lamour et al. [3] improved the hysteresis characteristics of GMR by pinning the direction of the layer. Shu-Hsien Liao et al. [4] reduced the hysteresis of TMR with an AC modulation magnetic field. Fei Xie, Roland Weiss, and Robert Weigel [5] used controlled current pulses to compensate for hysteresis.

To reduce the characteristics of hysteresis of TMR in practical application, the essential work should be the analysis and mathematical description of hysteresis characteristics. The original research on hysteresis characteristics focused on the physical model, such as the Stoner–Wohlfarth model [6]. The physical model needs deep expert knowledge on the inherent aspects of TMR and is difficult for regular researchers. The other kind of model is the phenomenon model. The phenomenon model constructs modeling hysteresis by studying the relationship between the input and output. The phenomenon model can be further classified into two types.

One type of phenomenon model is the black-box model based on a statistical model or machine learning model. This method is relatively simple to implement and does not need to pay attention to the explanation. However, this method has little apparent explanation, requires a large amount of data for training, and has stability problems and high hardware requirements to implement [7,8]. The other type of phenomenon model is the white-box method, which tries to describe the characteristics of the hysteresis based on a particular equation. This method has two primary kinds. One is based on the differential equation. It uses differential equations to describe the relationship between the input and output. Such as the Bouc-Wen model [9,10,11,12]. The other is operator-based equations. Examples include the Preisach model, KP (Krasnosel’skii-Pokrovskii) model, etc. The Preisach model is a simple, effective, and widely used model. Some researchers intend to extern its scope of application. Refs. [13,14,15,16] focused on the dynamic hysteresis model, and [17] focused on the vector hysteresis model. Some researchers try to apply it in practice. For example, ref. [18] used First Order Reversal Curve (FORC) to analyze the features of magnetic tunnel junction (MTJ) thin-film stacks. Ref. [19] used Preisach theory in reproducing the magnetization processes of the transformer core. Refs. [20,21,22] focus on the inverse model to compensate for the hysteresis. Some researchers try to improve its accuracy. Ref. [23] proposed a polynomial model that Prandtl-Ishlinskii (PI) operator can describe. Ref. [24] used a modified reduced Preisach model based on discrete empirical interpolation method (DEIM) to resolve the contradiction between the model accuracy and the number of the hysteresis kernels. Ref. [25] proposed an input-dependent Preisach model. This model is also called the nonlinear Preisach model. [26] identified the parameters of the Preisach model by the analysis method. Refs. [27,28] identified the parameters of the Preisach model by the machine learning method.

Although the Preisch model-based methods have been widely adopted to describe and analyze the characteristics of the hysteresis of TMR, these methods are either complicated or require more data. Therefore, based on the Preisach model, we explores the input and output relationship of the TMR sensor from the perspective of probability, and proposes a static hysteresis model based on probability; this model has higher accuracy than the classical Preisach model, especially in the case of a high-order rotation curve. Two kinds of sensors are tested.

The rest of this paper is organized as follows: Section 2 introduces the basic principle of the Preisach model. Section 3 details the proposed probability model. Section 4 presents experimental results and discussion. Finally, Section 5 concludes this work.

## 2. Preisach Introduction

**Figure 1:**
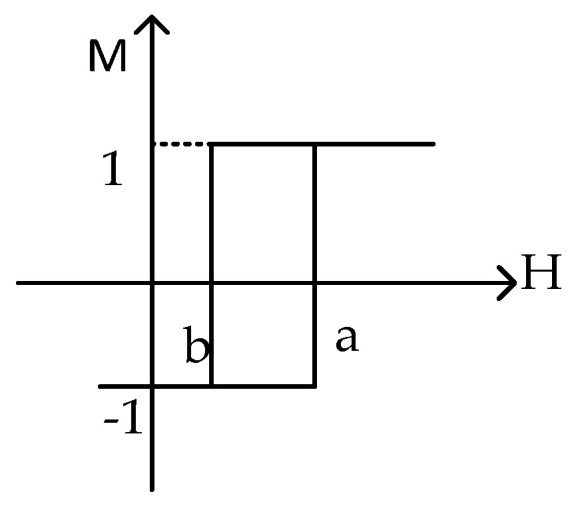
Preisach operator.

In this section, the Preisach model is introduced based on the literature [6]. The magnet is considered to be composed of finite simple hysteresis operators *γ_αβ_*, shown in Figure 1. The values of the operator can only be −1 or +1. The simple hysteresis operator has two switch values, the up-switch value *α*, and down-switch value *β*, with *α* ≥ *β*. The value of the operator is 1 when the input *H* ≥ *α* and 0 when the input *H* ≤ *β*; otherwise, it shows no change. In mathematical terms, it can be represented by Equation (1).

(1) $$\gamma_{\alpha \beta }=\{\begin{array}{c} 1\;\;\;\;\;\;\;\;\;\;\;\;\;\;\;\;H>\alpha \\ maintain\;value\;\;\;Other\\ -1\;\;\;\;\;\;\;\;\;\;\;\;\;\;\;\;H<\beta \end{array}.$$

The Preisach model can be represented as

(2) $$f\left(t\right)=\iint_{\alpha \leq \beta }^{}u\left(\alpha ,\beta \right)\gamma_{\alpha \beta }U\left(t\right)d\alpha d\beta .$$

where *U*(*t*) is the input, *γ_αβ_U*(*t*) is the value of the operators, and u(α,β) is the Preisach function.

**Figure 2:**
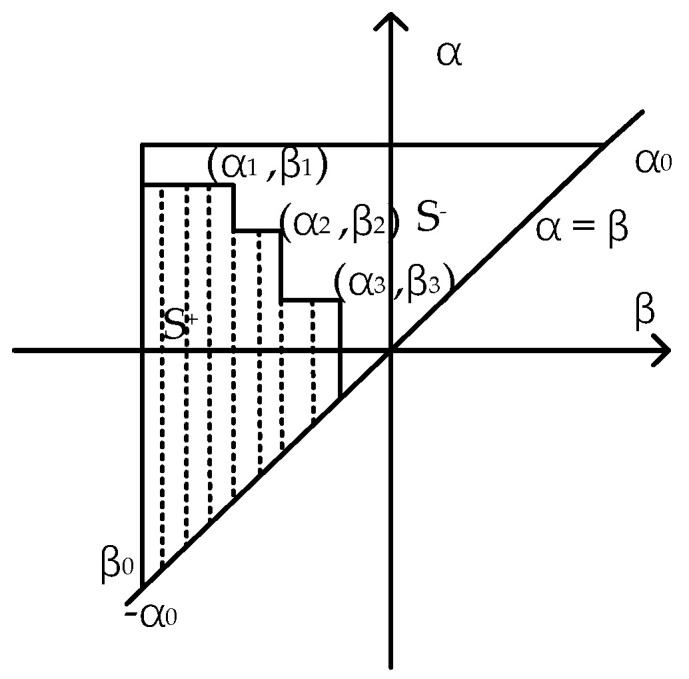
The geometric interpretation of the Preisach model.

The geometric interpretation of the Preisach model is shown in Figure 2. The triangle *T* is composed of *α = β*, *α = α*_0_, *β =* *β*_0_. The value of *u*(*α*,*β*) is non-zero inside the triangle *T* and zero outside the triangle *T*. A line parallel to the *β-*axis moves from *α =* *β*_0_ to *α = α*_1_ when the input monotonically increases from *β*_0_ to *α*_1_. Correspondingly, the value of all operators swept by the line becomes 1. A vertical line moves from *β = α*_1_ to *β =* *β*_1_ when the input monotonically decreases from *α*_1_ to *β*_1_. Correspondingly, the value of all operators swept by the line becomes −1. After a serial change of *β*_0_,*α*_1_,*β*_1_,*α*_2_,*β*_2_,*α*_3_,*β*_3_*,* the staircase line in the figure is formed. The points (*α*_1_,*β*_1_), (*α*_2_,*β*_2_) and (*α*_3_,*β*_3_) are reversal points. The staircase line separates the triangle *T* into two parts, *S*^+^ and *S*^−^. The values of the points in *S*^+^ are 1 and in *S*^−^ are −1.

The limiting ascending branch *f*^+^ (*α*) is formed by the monotonically increased input from negative saturation to some value *α*. The limiting descending branch *f*^−^ (*β*) is formed by the monotonically decreased input from positive saturation to some value *β*.

The first-order descending curve *f*^+^ (*α*,*β*) is formed by a monotonically decreased input following the limiting ascending branch *f*^+^ (*α*). The first-order ascending curve *f*^−^ (*α*,*β*) is formed by a monotonically increased input following the limiting descending branch. The high-order curve can then be defined accordingly.

In the Preisach model, has

(3) $$f^{+}\left(\alpha \right)=-f^{-}\left(-\alpha \right).$$

(4) $$f^{+}\left(\alpha ,\;\beta \right)=-f^{-}\left(-\beta ,-\alpha \right).$$

Equation (3) shows that the limiting ascending branch and the descending branch are odd-symmetric. Equation (4) shows that the first-order ascending curve and the descending branch are symmetric about *α = −β*.

There are two essential properties of the Preisach model, the wipe-out property and the congruency property. By the wipe-out property, only the alternating series of dominant input extrema are stored by the Preisach model, while all other input extrema are wiped out. By the congruency property, all minor hysteresis loops corresponding to back-and-forth variations of inputs between the same two consecutive extremum values are congruent. In the non-linear model, the congruency property becomes the property of equal vertical chords. All minor loops resulting from back-and-forth input variations between the same two consecutive extrema have equal vertical chords (output increments) for the same input values.

Equations (5) and (6) are used to calculate the output in the Preisach model. Equation (5) is used when the last stage is monotonically decreased, and Equation (6) is used when the last stage is monotonically increased.

(5) $$f\left(t\right)=-f^{+}+\overset{n-1}{\underset{k=1}{\sum }}\left(f_{M_{k}m_{k}}-f_{M_{k}m_{k-1}}\right)+f_{M_{k}u\left(t\right)}-f_{M_{k}m_{n-1}},$$

(6) $$f\left(t\right)=-f^{+}+\overset{n-1}{\underset{k=1}{\sum }}\left(f_{M_{k}m_{k}}-f_{M_{k}m_{k-1}}\right)+f_{-m_{n-1}}-f_{m_{n-1},-u\left(t\right)},$$

where *f*(*t*) is the output, *f^+^* is the output of the positive saturation, $f_{M_{k}m_{k}}$ is the first-order curve values of reversal points and $f_{-m_{n-1}}$ is the limiting curve value.

## 3. Probability Model

**Figure 3:**
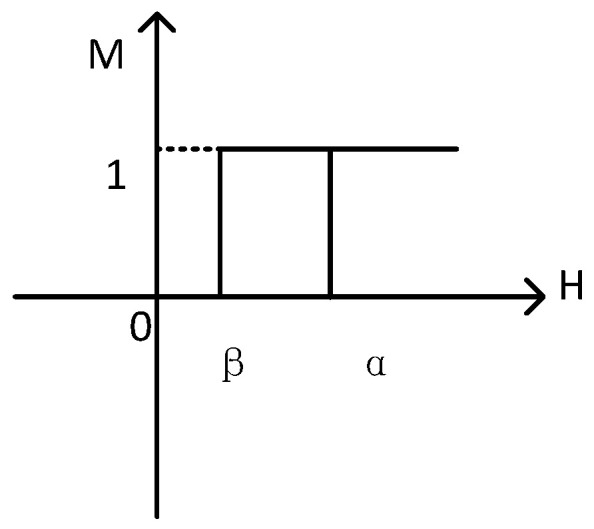
The hysteresis operator of the probability model.

Suppose that the magnet consists of a finite simple hysteresis operator; then, the hysteresis operator is shown in Figure 3.

The operator has two values, 0 and 1, while the up- and down-switch values are α and β. The operator can be represented by Equation (7):

(7) $${\gamma^{\prime }}_{\alpha \beta }=\{\begin{array}{c} 1\;H\geq \alpha \\ maintain\;value\;other\\ 0\;H\leq \beta \end{array}.$$

The output can be written as

(8) $$h\left(t\right)=\iint_{\beta \leq \alpha }^{}u\left(\alpha ,\beta \right){\gamma^{\prime }}_{\alpha \beta }U\left(t\right)d\alpha d\beta ,$$

where *γ^′^*_αβ_*U*(*t*) is the value of the operator, which can be 0 or 1, and *u*(*α*,*β*) is the power function; therefore, *h*(*t*) is the number of operators for which the value is 1. *h*(*t*) needs to be obtained using Equation (9) because the output of the sensor has negative values.

(9) $$h\left(t\right)=f\left(t\right)-f\left(-H_{sat}\right),$$

where *f*(*t*) is the experiment datum, *−H*_sat_ is the negative saturation input and *+H*_sat_ is the positive saturation input. When the input is smaller than the negative saturation, all of the operators are 0 and *h*(*t*) = 0. When the input is bigger than the positive saturation, all of the operators are 1 and *h*(*t*) = *M*, where *M* is the number of operators.

Let

(10) $$g\left(t\right)=\frac{h\left(t\right)}{M}.$$

We know that *h*(*t*) is the number of the operators for which the value is 1, and *M* is the number of operators. Therefore, *g*(*t*) is the probability of the operators having a value of 1. Suppose the up-switch value and down-switch value of the operator are random variables, *A* and *B*, then we obtain 3.

(11) $$A\in \left[-H_{sat},+H_{sat}\right].$$

(12) $$B\in \left[-H_{sat},+H_{sat}\right].$$

According to the probability theory,

(13) P(A=a)=P{α|α≤ a}.

(14) P(B=b)=P{β|β≤b}.

where *α* and *β* are the up-switch value and down-switch value of the operators. When the input monotonically increases from some value below the negative saturation to some value *a*, the limiting ascending curve *g*(*a*) is formed. From now on, if *g*(*t*) is not specified, it starts from negative saturation. All of the operators are 0 when the input is smaller than the negative saturation. Then, the operators with up-switch values *α* smaller than *a* become 1, when the input monotonically increases to *a*. At this time, all of the operators with a value of 1 have up-switch values smaller than *a*. Therefore, *g*(*a*) is the probability of the operators with up-switch values smaller than *a*. This can be written as Equation (15):

(15) g(a)=P(A=a).

When the input is monotonically decreased from some value above the positive saturation to some value *b*, the limiting descending curve *g*^−^(*b*) is formed. All of the operators are 1 when the input is more significant than the positive saturation. Then, the operators with down-switch values bigger than *b* become 0, as the input monotonically decreases to *b*. This means that the operators with a value of 1 are those with a down-switch value smaller than *b*. Therefore, *g*^−^(*b*) is the probability of the operators having down-switch values smaller than *b*. We can then write

(16) $$g^{-}\left(b\right)=P\left(B=b\right).$$

The first-order decreased curve *g*(*a*,*b*) is formed when *g*(*a*) is followed by a subsequent monotonically decreasing to *b*. Therefore, *g^−^*(*b*) can be considered *g*(*Hmax*,*b*). All operators with a value of 1 at *g*(*a*) have up-switch values smaller than *a*. Among these operators, those with down-switch values bigger than *b* become 0, as in *g*(*a*,*b*). Therefore, the operators with a value of 1 have up-switch values smaller than *a* and down-switch values smaller than *b*. We then obtain

(17) g(a, b)=P(A=a,B=b).

The first-order increased curve *g*^−^(*b*,*a*) is formed when the input monotonically increases to b after *g*^−^(*b*). Therefore, *g*(*a*) can be thought of as *g^−^* (−*Hmax*,*a*). All of the operators with a value of 1 at *g*^−^(*b*) have down-switch values smaller than *b*. This means that the operators with down-switch values bigger than *b* should be 0. As *g*^−^(*b*,*a*), the operators with up-switch values smaller than *a* in the 0-value operators turn to 1. Therefore,

(18) $$\begin{array}{c} g^{-}\left(b,a\right)=P\left(B=b\right)+P\left(A=a,B>b\right)\\ =P\left(B=b\right)+P\left(A=a\right)-P\left(A=a,B=b\right)\\ =P\left(B+A\right).\;\;\;\;\;\;\; \end{array}$$

Equation (17) can be written as

(19) g(a,b)=P(A=a,B=b)=P(A=a)−(P(A=a)−P(A=a,B=b).

We can see that the increase in *g*^−^(*b*,*a*) is equal to the decrease in *g*(*a*,*b*). In addition, we obtain Equation (20),

(20) $$g\left(a,b\right)+g^{-}\left(b,a\right)=P\left(A=a\right)+P\left(B=b\right)=g\left(a\right)+g^{-}\left(b\right).$$

Let *a = −b*; then,

(21) $$\begin{array}{c} g\left(-b,b\right)+g^{-}\left(b,-b\right)=g\left(-b\right)+g^{-}\left(b\right)\\ =\frac{h\left(-b\right)}{h\left(Hsat\right)}+\frac{h^{-}\left(b\right)}{h\left(Hsat\right)}\\ =\frac{f\left(-b\right)-f\left(-Hsat\right)}{f\left(Hsat\right)-f\left(-Hsat\right)}+\frac{f^{-}\left(b\right)-f\left(-Hsat\right)}{f\left(Hsat\right)-f\left(-Hsat\right)}. \end{array}$$

If

(22) $$f\left(-b\right)=-f^{-}\left(b\right)$$

then, Equation (21) is equal to 1.

The second-order curve *g*(*a*_1_,*b*_1_,*a*_2_) is formed by monotonically increasing the input from *b*_1_ to *a*_2_ in the first-order curve *g*(*a*_1_,*b*_1_). As previously discussed, in the circumstance of first-order curve *g*(*a*_1_,*b*_1_), the values of operators with up-switch values smaller than *a*_1_ and down-switch values smaller than *b*_1_ are 1. As the input increases, the curve is changed to the seconde-order *g*(*a*_1_,*b*_1_,*a*_2_), and the values of operators with up-switch values smaller than *a*_2_ used to be 0 are changed to 1.

Let *A*_1_ represent the event {*α <* *a*_1_}, *B*_1_ represent the event {*β <* *b*_1_} and *A*_2_ represent the event {*α < a*_2_}. Then, we obtain

(23) $$g\left(a_{1},b_{1},a_{2}\right)=P\left(A_{1}B_{1}+A_{2}\right)=P\left(A_{1}B_{1}\right)+P\left(A_{2}\right)-P\left(A_{1}B_{1}A_{2}\right)$$

If *a*_2_ > *a*_1_, then

(24) $$g\left(a_{1},b_{1},a_{2}\right)=P\left(A_{1}B_{1}\right)+P\left(A_{2}\right)-P\left(A_{1}B_{1}\right)=P\left(A_{2}\right).$$

This is in accordance with the wipe-out property.

If *a*_2_ < *a*_1_, then

(25) $$g\left(a_{1},b_{1},a_{2}\right)=P\left(A_{1}B_{1}\right)+P\left(A_{2}\right)-P\left(A_{2}B_{1}\right)=g\left(a_{1},b_{1}\right)+g\left(a_{2}\right)-g\left(a_{2},b_{1}\right).$$

This means that the output increase from *g*(*a*_1_,*b*_1_) to *g*(*a*_1_,*b*_1_,*a*_2_) equals the output decrease from *g*(*a*_2_) to *g*(*a*_2_,*b*_1_).

The third-order curve *g*(*a*_1_,*b*_1_,*a*_2,_*b*_2_) is formed by the input monotonically decreasing from *a*_2_ to *b*_2_ after *g*(*a*_1_,*b*_1_,*a*_2_) (obviously, *a*_2_ < *a*_1_). Let *B*_2_ represent the event {*β <* *b*_2_}.

(26) $$g\left(a_{1},b_{1},a_{2},b_{2}\right)=P\left(\left(A_{1}B_{1}+A_{2}\right)\cap^{\;}B_{2}\right)=P\left(A_{1}B_{1}B_{2}\right)+P\left(A_{2}B_{2}\right)-P\left(A_{1}B_{1}A_{2}B_{2}\right).$$

If *b*_1_ > *b*_2_, then

(27) $$g\left(a_{1},b_{1},a_{2},b_{2}\right)=P\left(A_{1}B_{2}\right)+P\left(A_{2}B_{2}\right)-P\left(A_{2}B_{2}\right)=P\left(A_{1}B_{2}\right)=g\left(a,d\right).$$

This satisfies the wipe-out propriety.

If *b*_1_ < *b*_2_, then

(28) $$\begin{array}{c} \;\;\;g\left(a_{1},b_{1},a_{2},b_{2}\right)=P\left(A_{1}B_{1}\right)+P\left(A_{2}B_{2}\right)-P\left(A_{2}B_{1}\right)\\ =P\left(A_{1}B_{1}\right)+P\left(A_{2}\right)-P\left(A_{2}B_{1}\right)-\left(P\left(A_{2}\right)-P\left(A_{2}B_{2}\right)\right)\\ =g\left(a_{1},b_{1},a_{2}\right)-\left(P\left(A_{2}\right)-P\left(A_{2}B_{2}\right)\right). \end{array}$$

This means that the change in the third-order curve equals that of the first-order curve. This is equivalent to the property of equal vertical chords in the non-linear model. As mentioned before, monotonically increasing to *a_n_* means adding the probability of the new event *A_n_ =* {*α <* *a_n_*} to the original event. When the input monotonically decreases to *b_n_*, the operators with values of 1 should meet the event *B_n_ =* {*β* < *b_n_*}. Thus, any order curve can express as

(29) $$g\left(a_{1},b_{1},\ldots ,a_{n},b_{n}\right)=P\left(\left(\left(\left(A_{1}B_{1}+A_{2}\right)B_{2}+\ldots \right)B_{n-1}+A_{n}\right)B_{n}\right)$$

(30) $$=P\left(\left\{\left(B_{n-1}+A_{n}\right)B_{n}\right\}\right).$$

In Equation (30), {(*B_n_*_−1_ + *A_n_*)*B_n_*} is short for (((*A*_1_*B*_1_ + *A*_2_)*B*_2_ *+ …*)*B_n_*_−1_ + *A_n_*)*B_n_* (from Equation (29)). Next, we prove that the wipe-out property and equal vertical chords property are also true for higher-order curves.

Suppose the 2n-order curve satisfies the two properties. Then, Equation (31) can be obtained:

(31) $$g\left(a_{1},b_{1},\ldots ,a_{n},b_{n},u\left(t\right)\right)=P\left(\left\{\left(B_{n-1}+A_{n}\right)B_{n}\right\}+U\right)$$

Then, the (2*n +* 1)-order curve can be written as Equation (32):

(32) $$\begin{array}{c} g\left(a_{1},b_{1},\ldots ,a_{n},b_{n},a_{n+1},u\left(t\right)\right)=P\left(\left\{\left(B_{n}+A_{n+1}\right)\right\}U\right)=P\left(\left\{\left(B_{n}U+A_{n+1}U\right)\right\}\right)\\ =P\left\{B_{n}U\right\}+P\left(A_{n+1}U\right)-P\left(\left\{B_{n}UA_{n+1}U\right\}\right). \end{array}$$

If *u*(*t*) *> b_n_*, then

(33) $$\begin{array}{c} g\left(a_{1},b_{1},\ldots ,a_{n},b_{n},a_{n+1},u\left(t\right)\right)\\ =P\left(\left\{B_{n}\right\}\right)+P\left(A_{n+1}U\right)-P\left(\left\{B_{n}A_{n+1}\right\}\right)\\ =P\left(\left\{B_{n}\right\}\right)+P\left(A_{n+1}\right)-P\left(\{B_{n}\}A_{n+1}\right)+P\left(A_{n+1}U\right)-P\left(A_{n+1}\right)\;\\ =P\left(\left\{B_{n}\right\}+A_{n+1}\right)-\left(P\left(A_{n+1}\right)-P\left(A_{n+1}U\right)\right)\;\;\;\;\;\;\;\;\;\;\;\;\;\;\;\;\\ =g\left(a_{1},b_{1},\ldots ,a_{n},b_{n},a_{n+1}\right)-\left(g\left(a_{n+1}\right)-g\left(a_{n+1},u\left(t\right)\right)\right).\;\;\;\; \end{array}$$

Equation (33) shows that the (*2n* + 1)-order curve meets the equal vertical chords property.

If *u*(*t*) < *b_n_*, then

(34) $$\begin{array}{c} g\left(a_{1},b_{1},\ldots ,a_{n},b_{n},a_{n+1},u\left(t\right)\right)=P\left(\left\{U\right\}\right)+P\left(A_{n+1}U\right)-P\left(\left\{UA_{n+1}\right\}\right)\\ =P\left(\left\{U+A_{n+1}U\right\}\right)\\ =P\left(\left\{A_{n}+A_{n+1}\right\}U\right)\;\\ =P\left(\left\{A_{n}\right\}U\right)\;\;\;\;\;\;\;\;\;\;\;\;\;\;\;\;\\ =g\left(a_{1},b_{1},\ldots ,a_{n},u\left(t\right)\right).\;\;\;\; \end{array}$$

This is in accordance with the wipe-out property.

The (2*n* + 2)-order is a monotonically increased curve and can be written as follows:

(35) $$\begin{array}{c} g\left(a_{1},b_{1},\ldots ,a_{n},b_{n},a_{n+1},b_{n+1},u\left(t\right)\right)\\ =P\left(\left\{\left(B_{n}+A_{n+1}\right)B_{n+1}\right\}+U\right)\\ =P\left(\left\{\left(B_{n}+A_{n+1}\right)B_{n+1}\right\}\right)+P\left(U\right)-P\left(\left\{\left(B_{n}+A_{n+1}\right)B_{n+1}\right\}U\right)\\ =P\left(\left\{\left(B_{n}+A_{n+1}\right)B_{n+1}\right\}\right)+P\left(U\right)-P\left(\left\{\left(B_{n}+A_{n+1}\right)B_{n+1}U\right\}\right)\\ =P\left(\left\{\left(B_{n}+A_{n+1}\right)B_{n+1}\right\}\right)+P\left(U\right)-P\left(\left\{\left(B_{n}U+A_{n+1}B_{n+1}U\right)\right\}\right)\\ =P\left(\left\{\left(B_{n}+A_{n+1}\right)B_{n+1}\right\}\right)+P\left(U\right)-P\left(\left\{\left(B_{n}U\right)\right\}\right)-P\left(A_{n+1}B_{n+1}U\right)\\ \;+P\left(\left\{\left(B_{n}UA_{n+1}\right)\right\}\right). \end{array}$$

If *u*(t) *< a_n_*_+1_,

(36) $$\begin{array}{c} g\left(a_{1},b_{1},\ldots ,a_{n},b_{n},a_{n+1},b_{n+1},u\left(t\right)\right)\\ =P\left(\left\{\left(B_{n}+A_{n+1}\right)B_{n+1}\right\}\right)+P\left(U\right)-P\left(B_{n+1}U\right)\\ =g\left(a_{1},b_{1},\ldots ,b_{n+1}\right)+g\left(u\left(t\right)\right)-g\left(u\left(t\right),b_{n+1}\right). \end{array}$$

The equal vertical chords property is thus proved.

If *u*(*t*) > *a_n+_*_1_, then

(37) $$\begin{array}{c} g\left(a_{1},b_{1},\ldots ,a_{n},b_{n},a_{n+1},b_{n+1},u\left(t\right)\right)\\ =P\left(\left\{\left(B_{n}+A_{n+1}\right)B_{n+1}\right\}\right)+P\left(U\right)-P\left(\left\{\left(B_{n}U\right)\right\}\right)-P\left(B_{n+1}A_{n+1}\right)\\ \;+P\left(\left\{\left(B_{n}A_{n+1}\right)\right\}\right)\;\;\;\;\\ =P\left(\left\{\left(B_{n}B_{n+1}+A_{n+1}B_{n+1}\right)\right\}\right)+P\left(U\right)-P\left(\left\{\left(B_{n}U\right)\right\}\right)-P\left(B_{n+1}A_{n+1}\right)\\ \;+P\left(\left\{\left(B_{n}A_{n+1}\right)\right\}\right)\;\\ =P\left(\left\{\left(B_{n}B_{n+1}\right)\right\}\right)+P\left(A_{n+1}B_{n+1}\right)-P\left(\left\{\left(B_{n}A_{n+1}B_{n+1}\right)\right\}\right)+P\left(U\right)\\ \;-P\left(\left\{\left(B_{n}U\right)\right\}\right)-P\left(B_{n+1}A_{n+1}\right)+P\left(\left\{\left(B_{n}A_{n+1}\right)\right\}\right)\\ =P\left(\left\{\left(B_{n}\right)\right\}\right)+P\left(U\right)-P\left(\left\{\left(B_{n}U\right)\right\}\right)\\ =P\left(\left\{\left(B_{n}\right)\right\}+U\right)\\ =g\left(a_{1},b_{1},\ldots b_{n},u\left(t\right)\right). \end{array}$$

This is in accordance with the wipe-out property. Therefore, the two properties are valid for any order curve. We can use these two properties to calculate any curve starting from the saturation input.

## 4. Experiment and Results

### 4.1. Experiment Introduction

**Figure 4:**
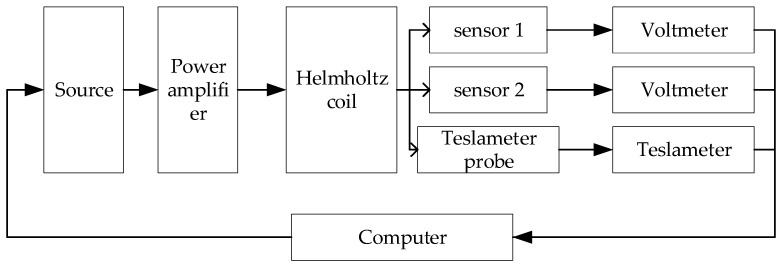
Experimental scheme.

The experimental scheme is shown in Figure 4. The high-precision power supply drives the Helmholtz coil through the power amplifier to generate a magnetic field. Sensor 1, sensor two, and Tesla meter probes are fixed in the uniform magnetic field area of the Helmholtz coil. Measure the magnetic field with a Tesla meter. Two high-precision voltmeters are used to measure the outputs of the sensors. The computer controls the whole acquisition process.

**Figure 5:**
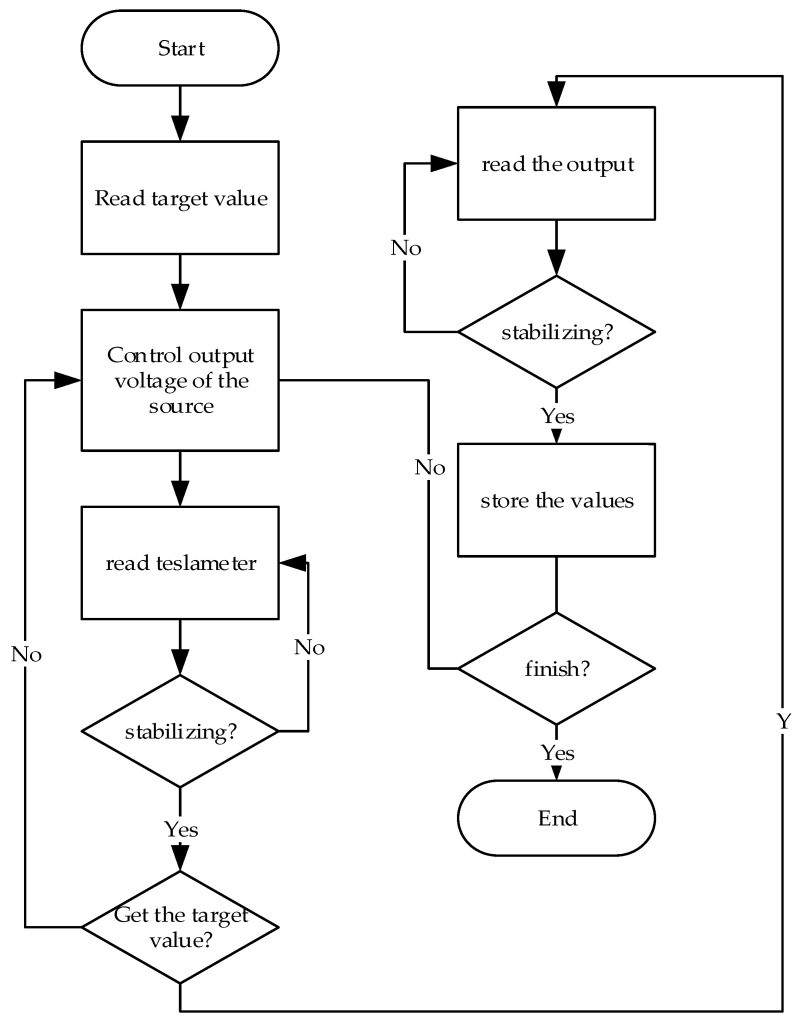
Flow chart.

The flow chart of acquisition is shown in Figure 5. First, the computer reads the required magnetic field, calculates an approximate voltage value, controls the high-precision power supply to output the voltage value, and collects the Tesla meter reading. Adjust the output voltage value according to the difference between the Tesla meter reading and the target value when the reading is stable. This operation continues until the target value is obtained. Then read the data of the two sensors, wait until the sensor data is sound, record the data, and then read the next target value.

### 4.2. Results and Discussion

**Figure 6:**
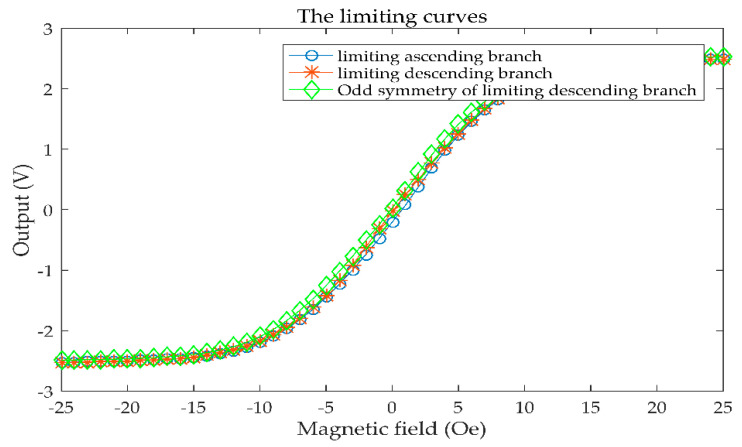
The limiting curves.

The limiting curves *f*(*u*(*t*)) acquired following the experiment are shown in Figure 6. fifty-one sample points were chosen, denoted as *f*(*a*_1_), *f*(*a*_2_), *…*, *f*(*a*_21_), and the odd symmetry of the limiting descending branch is also plotted in Figure 6 to study the symmetry of the limiting curves.

**Figure 7:**
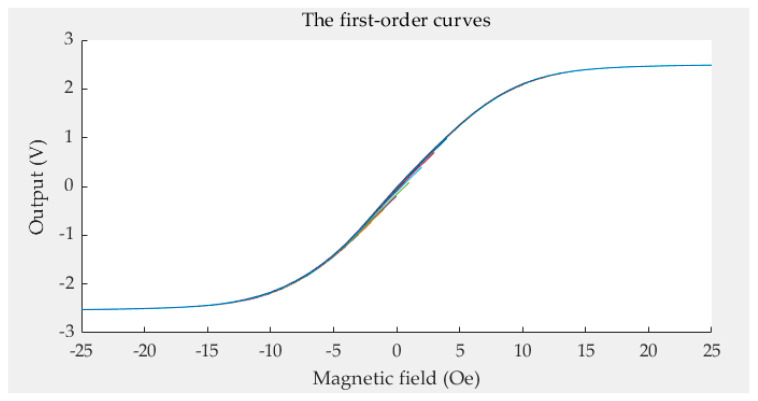
The first-order curves.

In Figure 6, the *y*-axis represents the sensor’s output, and the *x*-axis represents the magnetic field. We can see that the odd symmetry of the descending branch is not identical to that of the ascending branch. This means that the limiting ascending curves and limiting descending curves are not symmetric. This might be due to the material and manufacturing process of the sensor. There are 50 first-order curves corresponding to 51 acquisition points. The first-order curves are shown in Figure 7. Every color curve represents a first-order curve.

According to the discussion in Section 3, we obtain Equation (38):

(38) $$g\left(u\left(t\right)\right)=\frac{h\left(u\left(t\right)\right)}{h\left(+\mathrm{Hsat}\right)}=\frac{f\left(u\left(t\right)\right)-f\left(-Hsat\right)}{f\left(Hsat\right)-f\left(-Hsat\right)}.$$

**Figure 8:**
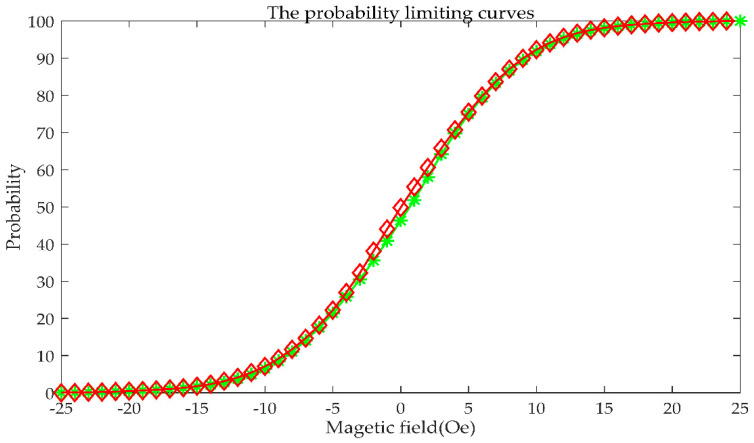
The limiting curve of the probability model.

**Figure 9:**
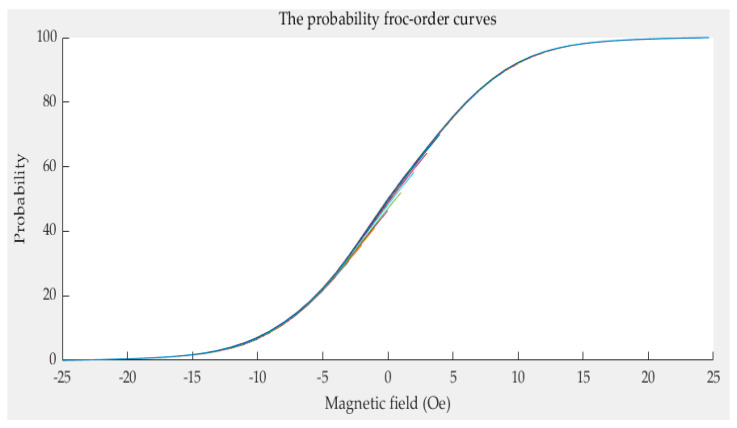
The first-order curves of the probability model.

The limiting curve *g*(*a*_i_) and several first-order curves *g*(*a*_i_,*b*_j_) can be obtained by Equation (38). The limiting curve *g*(*a*_i_) is shown in Figure 8 and the first-order curves *g*(*a*_i_,*b*_j_) are shown in Figure 9. Every color curve represents a first-order curve.

**Figure 10:**
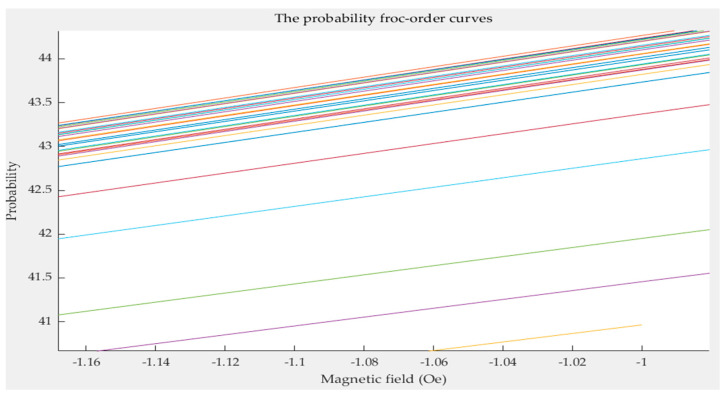
A partial enlarged view of the first-order curves.

Any limiting ascending curve *g*(*a*) can be obtained by linearly interpolating *g*(*a_i_*). Then, any first-order curve *g*(*a*,*b*) needs to be calculated. One method is to directly interpolate the first-order curves *g*(*a_i_*,*b_j_*). However, because the distance between the first-order revolution curves does not have apparent characteristics, as shown in Figure 10, the accuracy of this method is not high.

Another method to calculate any first-order curve *g*(*a*,*b*) is as follows. Any first-order curve *g*(*a*,*b*) represents the probability of up-switch values smaller than *a* and down-switch values smaller than *b*. This can be written as Equation (39):

(39) g(a,b)=P(A=a,B=b).

According to the property of probability, Equation (40) can be written as

(40) g(a,b)=P(A=a,B=b)=P(B=b|A=a)P(A=a).

**Figure 11:**
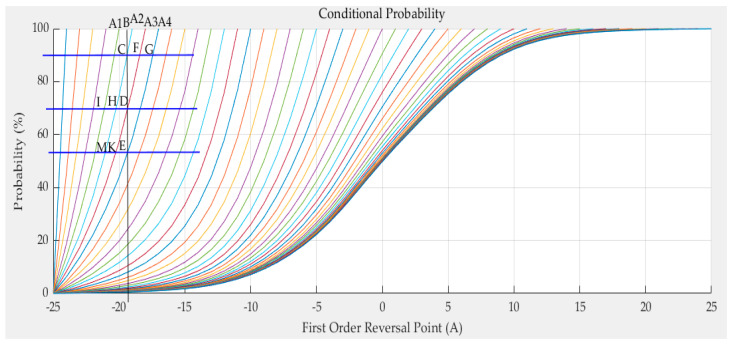
The conditional probability curves.

In Equation (40), *P*(*A*) can be obtained by interpolating *g*(*a*_i_). *P*(*A*,*B*) can be calculated when *P*(*B|A*) is determined. Then, experimental *P*(*B_j_|A_i_*) is needed and *P*(*A_i_*,*B_j_*) are obtained. Therefore, we can obtain the conditional probability using Equation (41). The conditional probability curves are shown in Figure 11.

(41) $$P(B_{j}|\;A_{i})=\frac{P\left(A_{i}=a_{i},B_{j}=b_{j}\right)}{P\left(A_{i}=a_{i}\right)}=\frac{g\left(a_{i},b_{j}\right)}{g\left(a_{i}\right)}.$$

In Figure 11, the *x* axis represents the magnetic field, and the *y* axis represents the probability. Every curve in the figure represents one conditional probability. The conditional probability curves intersect the line at *y* = 100% at the reversal points, such as *A*_1_(*a*_1_,1), *A*_2_(*a*_2_,1), *A*_3_(*a*_3_,1), and *A*_4_(*a*_4_,1). Some features can be seen in the figure. The probability is 0 at negative saturation and 1 when the magnetic saturation is more significant than the reversal points. The probability slope with more minor reversal points is more significant than that with more prominent reversal points—the greater the reversal points, the denser the curves. The spaces between the curves decrease with the increase in the reversal points. The ratio of the spacing between adjacent curves remains roughly unchanged. For example, the ratio of *A*_2_*A*_3_ and *A*_3_*A*_4_ is roughly equal to the ratio of *CF* and *FG*; this can be written as Equation (42):

(42) $$\frac{CF}{FG}=\frac{A_{2}A_{3}}{A_{3}A_{4}}.$$

Next, we illustrate how to calculate *P*(*B|A*). As shown in Figure 10, the straight line *x = b* intersects curves *A*_2_*C*, *A*_3_*D* and *A*_4_*E* at the points *C* (*b*,*P*(*b|a*_2_)), *D* (*b*,*P*(*b|a*_3_)) and *E* (*b*,*P*(*b|a*_4_)), respectively. Suppose the probability of point *D* is needed. This means that curves *A*_2_*C* and *A*_4_*E* are known; however, curve *A*_3_*D* is not. The probabilities of points *C* and *E* can be calculated by interpolating the curves *A*_2_*C* and *A*_4_*E*, respectively. Then, according to point C’ and E’s probability, the abscissa g and m of point G and point M are obtained by interpolating curve A4E and curve A2M respectively. According to the previous assumptions, the lengths of EK and CF can be obtained. Moreover, the probability of *P*(*B|A*_3_) = *P*(*b|a*_3_) needs to be calculated. It can be supposed that curve *KF* is straight line. Then, the triangle *CFD* is similar to triangle *DEK*. Therefore, the following equation can be obtained:

(43) $$\frac{CD}{DE}=\frac{CF}{KE}=\frac{CG*\frac{A_{2}A_{3}}{A_{2}A_{4}}}{ME*\frac{A_{3}A_{4}}{A_{2}A_{4}}}=\frac{CG*A_{2}A_{3}}{ME*A_{3}A_{4}}.$$

Substitute the coordinate value to Equation (43); then, we obtain

(44) $$\frac{P(b|\;a_{2})-P(b|\;a_{3})}{P(b|\;a_{3})-P(b|\;a_{4})}=\frac{b-g*\left(a_{2}-a_{3}\right)}{\left(m-b\right)*\left(a_{3}-a_{4}\right)}.$$

Then, *P*(*b|*$a_{3}$) can be calculated by Equation (45):

(45) $$P(b|\;a_{3})=\frac{\left(m-b\right)*\left(a_{3}-a_{4}\right)P(b|\;a_{2})+\left(m-b\right)*\left(a_{3}-a_{4}\right)P(b|\;a_{4})}{(b-g)*\left(a_{2}-a_{3}\right)+\left(m-b\right)*\left(a_{3}-a_{4}\right)}.$$

If the point *C* (*b*,*P*(*b|a*_2_)) is needed, then curves *A*_1_*H* and *A*_3_*D* are known and curve *A*_2_*C* is not. The triangle *BCA*_2_ is approximately similar to triangle *BDA*_3_. Therefore,

(46) $$\frac{BC}{CD}=\frac{BA_{2}}{HD}=\frac{BA_{2}}{ID*\frac{A_{2}A_{3}}{A_{1}A_{3}}}.$$

Substitute the coordinate value to Equation (46); then,

(47) $$\frac{1-P(b|\;a_{2})}{P(b|\;a_{2})-P(b|\;a_{3})}=\frac{b-a_{2}}{\left(i-b\right)*\frac{a_{2}-a_{3}}{a_{1}-a_{3}}}.$$

Then,

(48) $$P(b|\;a_{2})=\frac{\left(i-b\right)\left(a_{2}-a_{3}\right)+\left(b-a_{2}\right)\left(a_{1}-a_{3}\right)P(b|\;a_{3})}{\left(b-a_{2}\right)\left(a_{1}-a_{3}\right)+\left(i-b\right)\left(a_{2}-a_{3}\right)}.$$

**Figure 12:**
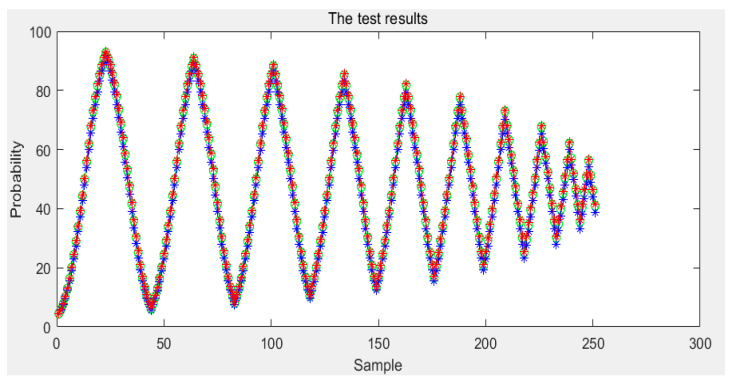
Test results of the first kind sensor.

**Figure 13:**
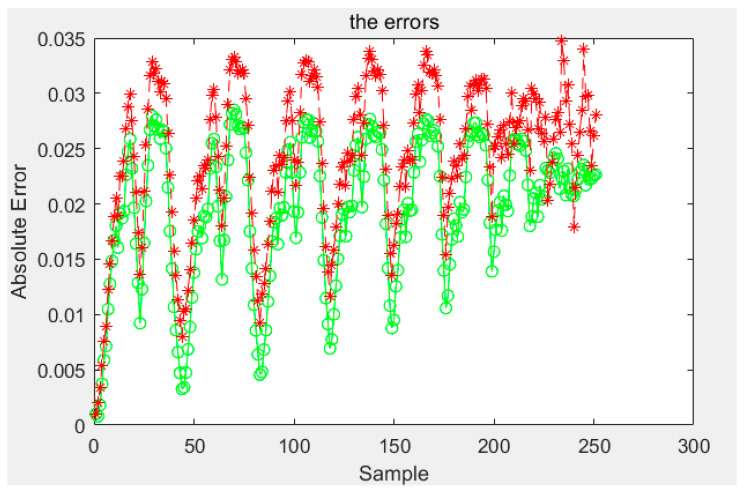
Errors of the first kind sensor.

**Figure 14:**
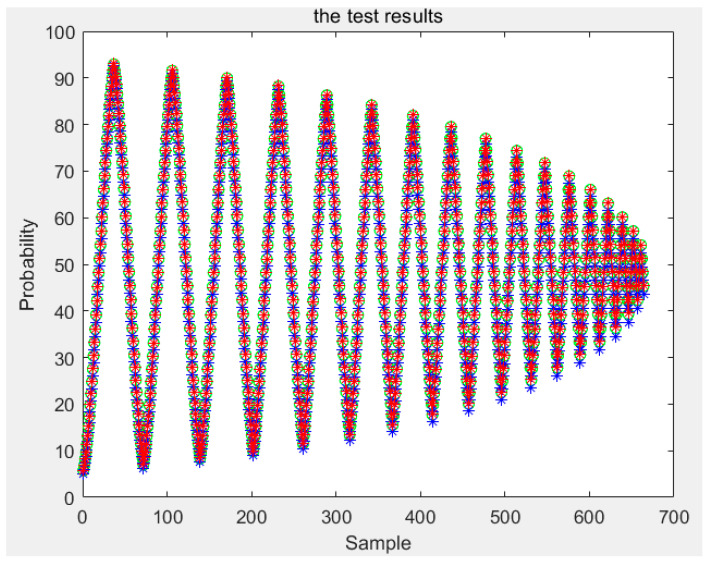
Test results of the second kind sensor.

**Figure 15:**
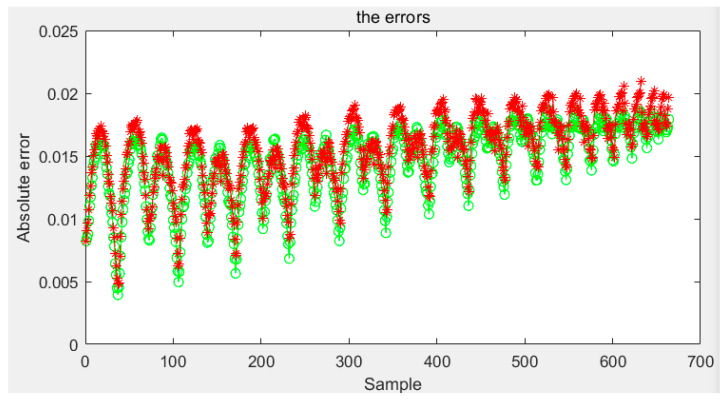
Errors of the second kind sensor.

According to the above, any conditional probability *P*(*B|A*) can be calculated. Moreover, any *P*(*A*) can be obtained by interpolating the limiting curve. Then, the probability *P*(*AB*) of any first-order reversal curve can be obtained and, according to Section 3, any order reversal curves can be predicted. In our experiment, two types of sensors are used. The test curves were acquired, and the probability model and the classical Preisach model were used to predict the curve. The results of the two models were compared with the experiment data; then, the experimental data and the results of the classical Preisach model were turned into probability data by Equation (38). The results of the first kind of sensor are shown in Figure 12. The first sensor’s error of the probability model and that of the classic Preisach model are shown in Figure 13, in which the *y* axis represents the probability, and its unit is 1. The results of the second sensor are shown in Figure 14. The error is shown in Figure 15, in which the *y* axis represents the probability, and its unit is 1.

The results show that the error of the probability model is clearly smaller than that of the classic Preisach model. At the beginning stage, the errors of the two models are similar. That is because the beginning stage is the limiting curve. Both models use the limiting curve linear interpolation method to predict the limiting curve. However, in the subse-quent data, the error of the probability model is less than that of the classical Preisach model, especially in the high-order reversal curve. It can be seen that in the last section of data, the error fluctuation range of classical Preisach tends to enlarge. In contrast, the error fluctuation of the probability model tends to narrow. Both models can predict the sensor’s output; however, the probability model is more accurate than the classic Preisach model.

Various indicators of error are shown in Table 1. show that the mean error of one sensor between the Preisach model and probability model are 0.0245 and 0.0197; the variance is 4.5650 × 10^−5^ and 4.1695 × 10^−5^, and the maximum error is 0.0347 and 0.0209. And, the mean error of the other sensor between the Preisach model and probability model are 0.0156 and 0.0146, respectively, the variance is 8.3547 × 10^−6^ and 8.2343 × 10^−6^, and the maximum error is 0.0209 and 0.0187. The reduced ratio is listed as the relevant index of the Preisach model minus the relevant index of the probability model and then divided by the appropriate index of the Preisach model. It can be seen that the lift of sensor 1 is greater than that of sensor 2. This is because the magnetic hysteresis of sensor 1 is relatively large relative to its range, while that of sensor 2 is relatively small relative to its range.

**Figure 16:**
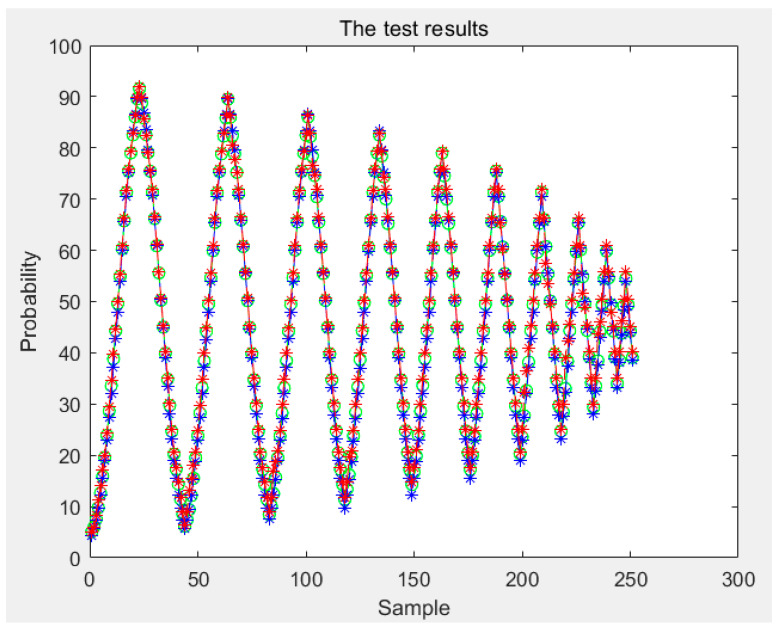
Test results of the first kind sensor for sparse data.

**Figure 17:**
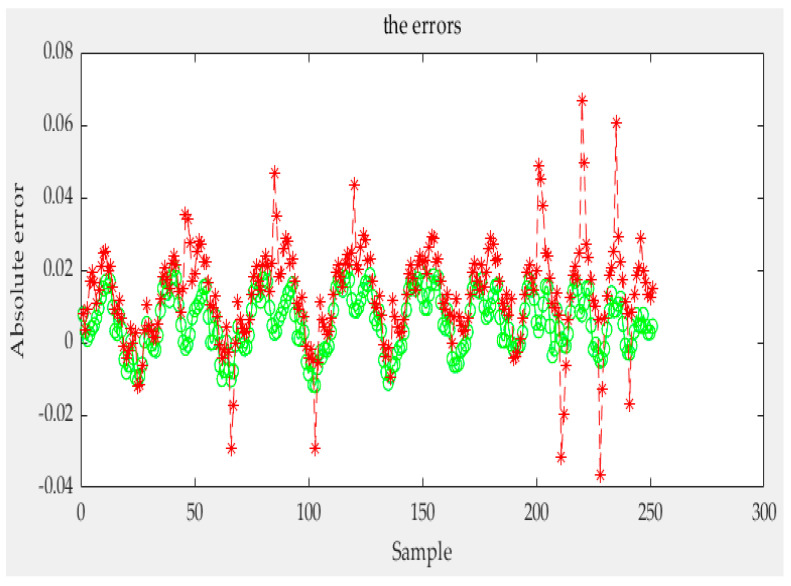
Errors of the first kind sensor for the sparse data.

**Figure 18:**
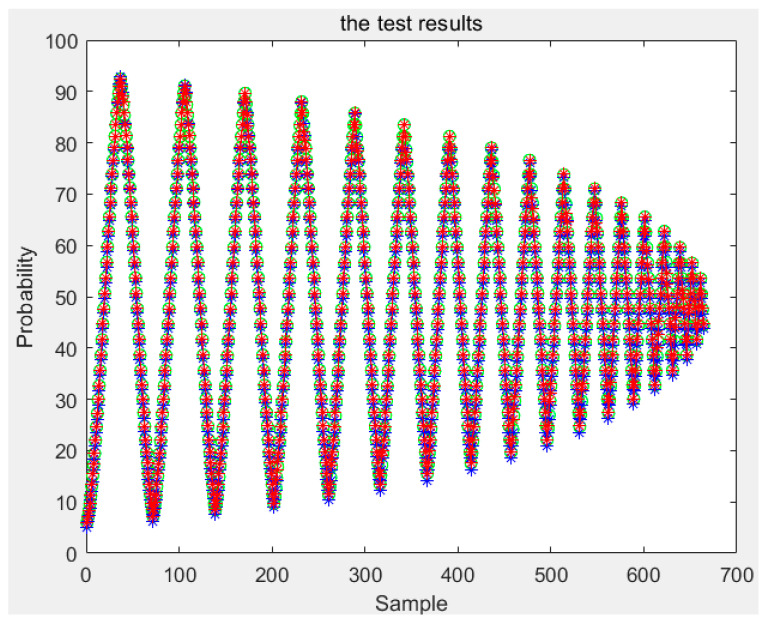
Test results of the second kind sensor for the sparse data.

**Figure 19:**
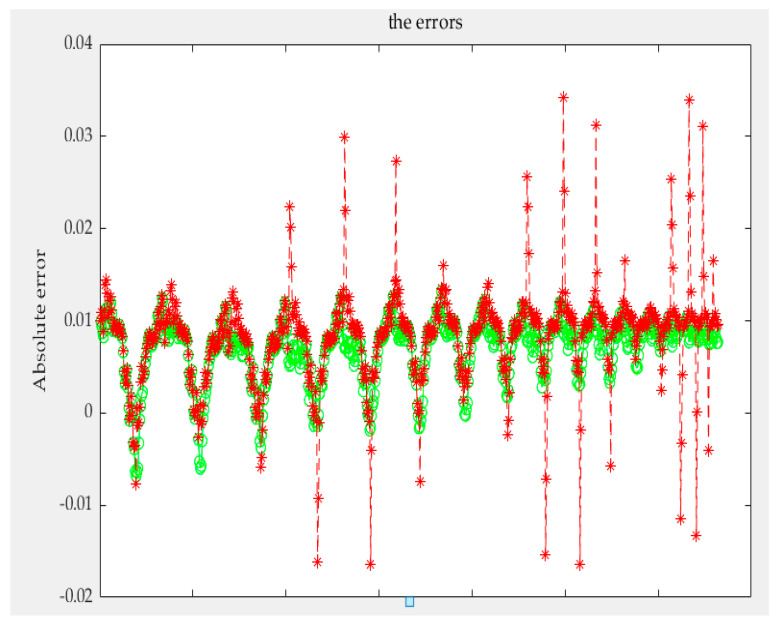
Errors of the second kind sensor for the sparse data.

The amount of data of the first-order reversal curve, that is, the number of reversal points, contradicts the the model’s accuracy. The model’s accuracy is higher with more reversal points, but the time required is significantly increased. With fewer turning points, the time required is less, but the accuracy may be reduced. So we also verified the model by using sparse data. This paper uses the first-order reversal curve data with an interval of 5 OE between reversal points to test two models and two sensors. The results and errors of sensor one are shown in Figure 16 and Figure 17, and the results and mistakes of sensor two are shown in Figure 18 and Figure 19.

The error indicators are shown in Table 2. The mean error of the two models on one sensor is 0.0135 and 0.0058, the variance is 1.7869 × 10^−4^ and 6.3849 × 10^−5^, and the maximum error is 0.0667 and 0.0204. On the other sensor, the mean error of the Preisach model and probability model are 0.0082 and 0.0070, respectively, the variance is 2.7725 × 10^−5^ and 1.1834 × 10^−5^, and the maximum error is 0.0342 and 0.0132. It can be seen that under the condition of using sparse data, the improvement of the probability model is more evident than that of the Preisach model. Especially in the case of a high-order reversal curve, the error of the Preisach model increases. In contrast, the error of the probability model hardly changes, and even the fluctuation range tends to narrow. Moreover, the complexity of the model and the required data of the probability model are approximately the same as the classic Preisach model.

## 5. Conclusions

This paper analyzes hysteresis from the perspective of probability and proposes a probability-based hysteresis modeling method. This method has the same complexity and required data level as the classical model but achieves higher accuracy. The test results show that the error of the probability model is smaller than that of the Preisach model, especially under the condition of a high-order reversal curve.

It can be seen that the error of the probability model relative to the Preisach model is reduced, especially in the case of a high-order reversal curve. The amount of the first-order reversal points is a primary reason affecting the accuracy of the model. This paper uses two steps, e.g., 1 OE and 5 OE to evaluate the two models, which shows that the probability model has higher accuracy than the classical model in sparse data, which shows that this method can effectively improve test efficiency while improving accuracy.

Furthermore, the reduction of the number of first-order reversal points will significantly shorten the acquisition time. The results show that the improvement of the probability model is more evident than that with a break of 1 OE. This helps to shorten the acquisition time. In addition, the congruence property of the Preisach model requires the consistency of small loops, while the probability model only requires the equality of vertical chords.

## Figures and Tables

**Table 1:** Errors.

Sensors	Models	Average Value	Reduced Proportion	Absolute Maximum	Reduced Proportion	Variance	Reduced Proportion
Sensor 1	Preisach model	0.0245	19.6%	0.0347	39.8%	4.5650 × 10^−5^	8.6%
	Probability model	0.0197		0.0209		4.169 × 10^−5^	
Sensor 2	Preisach model	0.0156	6.5%	0.0209	10.5%	8.3547 × 10^−6^	14.4%
	Probability model	0.0146		0.0187		8.2343e × 10^−6^	

**Table 2:** Errors.

Sensors	Models	Average Value	Reduced Proportion	Absolute Maximum	Reduced Proportion	Variance	Reduced Proportion
Sensor 1	Preisach model	0.0135	57.0%	0.0667	69.4%	1.7869 × 10^−4^	64.3%
	Probability model	0.0058		0.0204		6.3849 × 10^−5^	
Sensor 2	Preisach model	0.0082	14.6%	0.0342	61.4%	2.7725 × 10^−5^	57.3%
	Probability model	0.0070		0.0132		1.1834 × 10^−5^	

## Data Availability

Not applicable.
